# Small airway dysfunction as predictor and marker for clinical response to biological therapy in severe eosinophilic asthma: a longitudinal observational study

**DOI:** 10.1186/s12931-020-01543-5

**Published:** 2020-10-21

**Authors:** Mustafa Abdo, Henrik Watz, Vera Veith, Anne-Marie Kirsten, Heike Biller, Frauke Pedersen, Erika von Mutius, Matthias V. Kopp, Gesine Hansen, Benjamin Waschki, Klaus F. Rabe, Frederik Trinkmann, Thomas Bahmer

**Affiliations:** 1 LungenClinic Grosshansdorf, Airway Research Center North (ARCN), German Center for Lung Research (DZL), Wöhrendamm 80, 22927 Grosshansdorf, Germany; 2 Pulmonary Research Institute At the LungenClinic Grosshansdorf, Airway Research Center North (ARCN), German Center for Lung Research (DZL), Grosshansdorf, Germany; 3 Dr. Von Hauner Children’s Hospital, Ludwig Maximilians University of Munich, Comprehensive Pneumology Center Munich (CPC-M), German Center for Lung Research (DZL), Munich, Germany; 4 Division of Pediatric Pulmonology and Allergology, University Children’s Hospital Luebeck, Airway Research Center North (ARCN), German Center for Lung Research (DZL), Luebeck, Germany; 5 Department of Paediatric Pneumology, Allergology and Neonatology, Hannover Medical School, Biomedical Research in Endstage and Obstructive Lung Disease (BREATH), German Center for Lung Research (DZL), Hannover, Germany; 6 Department of General and Interventional Cardiology, University Heart Center Hamburg, Hamburg, Germany; 7 Department of Respiratory and Critical Care Medicine, Thoraxklinik, University of Heidelberg, Translational Lung Research Center Heidelberg (TLRC), German Center for Lung Research (DZL), Heidelberg, Germany; 8 Dept for Internal Medicine I, University Hospital Schleswig-Holstein, Campus Kiel, Airway Research Center North (ARCN), German Center for Lung Research (DZL), Kiel, Germany

**Keywords:** Anti-T2 biologics, Asthma control, Small airways dysfunction, FEV1

## Abstract

**Background:**

Anti-T2 biological therapies have proven to effectively reduce acute exacerbations and daily doses of oral steroids in severe eosinophilic asthma. Despite the remarkable clinical efficacy, there are usually only moderate improvements in airflow limitation, suggesting that other measures of lung function like small airway dysfunction (SAD) might better reflect the clinical response. We aimed to investigate if measures of small airway function would predict and correlate with the clinical response to anti-T2 therapy.

**Methods:**

We studied data of patients who were previously included in the German prospective longitudinal All Age Asthma Cohort (ALLIANCE) that recruits asthma patients of all severity grades and inflammatory phenotypes. The selection criteria for this analysis were adult patients with severe eosinophilic asthma under treatment with anti-T2 biological agents. Asthma control was assessed by asthma control test (ACT) and number of severe exacerbations. Small airway function was assessed by the frequency dependence of resistance (FDR, R5-20)) derived from impulse oscillometry (IOS) and the mean forced expiratory flow between 25 and 75% of the forced vital capacity (FEF_25-75_). We also studied air trapping (RV and RV/TLC), blood eosinophils and FeNO. Patients were classified into responders and partial or non-responders. Clinical response was defined as at least 50% reduction in annualized severe exacerbations and daily oral steroid doses accompanied with a minimum increase of 3 points in the ACT score. We used a Receiver Operator Characteristic (ROC) to study the capacity of FDR in predicting clinical response compared to other clinical variable like blood eosinophils. We studied the correlation between FDR measures and clinical response, represented by the ACT score and number of exacerbations, using linear regressions.

**Results:**

20 patients were included (mean age, 59 ± 9 years; 60% female; mean body mass index (BMI), 27.6 ± 5.4 kg/m^2^; mean absolute blood eosinophils, 570 ± 389/µl; mean number of severe exacerbations 12 months prior to initiating the biological therapy, 5.0 ± 3; mean predicted FEV1, 76 ± 21%; mean predicted FDR, 224 ± 140%; mean daily prednisolone dose, 6.4 ± 4.9 mg; mean ACT score, 15 ± 5). Responders had significantly higher baseline FDR compared to partial or non-responders but similar FEV1, FEF_25–75,_ RV and RV/TLC. ROC analysis showed that the combination of FDR and blood eosinophils had the best predictive capacity of the clinical response among all tested clinical markers (FeNO, FEV1, FDR, blood eosinophils) with an AUC of 85% [67–100%], (CI = 0.95, p = 0.01). Linear regressions indicated better associations between improvements in FDR and ACT score (R^2^ = 0.42, p = 0.001) than with FEV1 and ACT score (R^2^ = 0.25, p = 0.013). Likewise, we observed better associations between improvements in FDR and reduction of exacerbations (R^2^ = 0.41, p = 0.001) than with FEV1 (R^2^ = 0.20, p = 0.025).

**Conclusion:**

Our data suggest that severe SAD may represent a distinct phenotype of eosinophilic asthma that substantially improves under anti-T2 biological therapy. Measures of small airway function might be useful in selecting appropriate patients qualifying for anti-T2 biological therapy in addition to blood eosinophil count.

**To the Editor,**

Anti-T2 biological therapy is recommended as an add-on treatment for severe eosinophilic asthma [1]. Several randomized clinical trials have demonstrated the efficacy of anti-T2 therapy in reducing acute exacerbations and daily doses of oral steroids, while also indicating some improvements in forced expiratory volume in 1 s (FEV1) [2–4]. The improvements in FEV1 do not seem to correspond properly with the marked clinical response to biological therapy, suggesting that measures of large airway obstruction like the FEV1 might be a poor tool and that measures of other lung function abnormalities like small airway dysfunction (SAD) might better reflect the clinical response. Indeed, SAD is a hallmark of asthma that is associated with disease severity and poor symptom control [5]. Moreover, SAD is associated with frequent exacerbations [6, 7] and has a negative impact on daily physical activity [8]. We aimed to investigate if measures of SAD in patients with severe eosinophilic asthma might better correlate or even be helpful in predicting clinical response to biological therapy, thereby potentially describing a distinct phenotype within this severe asthma population.

In this study, we analyzed data of patients who were previously recruited in the prospective longitudinal All Age Asthma Cohort (ALLIANCE), a national cohort of pediatric and adult patients with asthma in Germany, initiated by the German Centre for Lung Research (DZL). The study was approved by the local ethics committee at the medical school Luebeck (Az.21–215) and is registered at clinicaltrials.gov (adult arm: NCT02419274) [9]. Since 2014, the adult arm of the ALLIANCE cohort recruits patients with mild to severe asthma and healthy controls. Patients had to be in specialist care for more than three months, and criteria of “difficult to control” asthma were addressed according to current guidelines [10]. Study visits take place in 12 months intervals. Patients had to have stable disease without acute exacerbations or respiratory tract infections within four weeks prior to study visits.

The selection criteria for this study were adult patients with severe eosinophilic asthma in whom a treatment with anti-T2 biological agent has been initiated while already being recruited into the observational ALLIANCE study and in whom at least one complete follow-up visit was available. Indication, prescription and administration of the biological therapy were not part of the study but rather were done by their respiratory physicians.

On each study visit, we assessed asthma control by asthma control test (ACT) as well as number of severe exacerbations 12 months prior to the study visit, defined as a burst of systemic corticosteroids for ≥ 3 days [10]. We performed a spirometry, body plethysmography and impulse oscillometry (Masterscreen Body and IOS, Vyaire Medical, Germany) according to guidelines [11–13]. IOS is a feasible diagnostic tool that measures increased resistance in peripheral airways at tidal breathing, even in subjects with normal spirometry [14]. We studied the small airway function using both spirometry measures (FEF_25–75_) and IOS measures (frequency dependence of resistance, FDR (R5Hz-R20Hz, kPa/l/s)). Percent predicted FDR values (FDR %pred.) were calculated according to recently provided prediction equations by the German KORA cohort [15]. We also included measures of air trapping like the residual volume (RV) and RV to total lung capacity ratio (RV/TLC) as indirect markers for SAD. We studied peripheral blood eosinophils and fractional exhaled nitric oxide (FeNo) as surrogates for eosinophilic airway inflammation [16, 17]. Based on their clinical response, patients were classified into responders and partial-/non-responders: Responders (n = 13) had at least a 50% reduction of severe exacerbations averaged over the last 12 months or ≥ 50% reduction in oral steroid doses [2, 18] and additionally, an increase in the ACT score by at least 3 points as this is the minimal clinically important difference (MCID) [19]. Partial-/Non-responders had less than 50% reduction in both severe exacerbations and in oral steroid doses and the ACT increase was beneath the MCID, or they even experienced worsening of symptoms (i.e. increase in severe exacerbations and a decrease in the ACT score) upon tapering systemic steroids. We evaluated the capacity of baseline FDR in predicting clinical treatment response by creating a Receiver Operator Characteristic (ROC). To evaluate FDR measures as potential markers for clinical response, we carried out comparative linear regressions between the change in FDR and other lung function measures with the change in severe exacerbations and in the ACT score.

Twenty patients with severe eosinophilic asthma under a treatment with anti-T2 agents (mepolizumab, n = 18; benralizumab, n = 1; dupilumab, n = 1) were included, (mean age, 59 ± 9 years; 60% female; mean body mass index (BMI), 27.6 ± 5.4 kg/m^2^). All patients were on inhaled corticosteroids (ICS; mean inhaled fluticasone equivalent 818 ± 403 µg) and long-acting β_2_ adrenoceptor agonists (LABA). 55% were treated with long-acting muscarinic receptor antagonists (LAMAs) and 80% were on maintenance oral corticosteroids (OCS, mean daily prednisolone dose 6.4 ± 4.9 mg) prior to starting with biological therapies. Mean predicted FEV_1_ at baseline was 76 ± 21%, mean FEF_25-75_ 1.1 ± 0.73 (l/s), mean predicted RV 148 ± 25%, mean RV/TLC 47 ± 8%, mean FDR 0.21 ± 0.18 kPa/L/s, mean FDR%pred. 224% ± 140%, mean absolute blood eosinophils 570 ± 389/µl, mean FeNO 60 ± 33 ppb. Mean ACT score was 15.5 ± 5.4 points and patients experienced 5 ± 3 severe exacerbations 12 months prior to initiating the biological therapy. Between responders and partial-/non-responder, there were no significant differences in the baseline values of all clinical variables except for FDR%pred, which was significantly higher in responders (Table 1). Area under the curve (AUC) for FDR%pred. was better than for FeNO, blood eosinophils, and FEV1 (Table 2). At a cut off of 191% for FDR%, we observed a sensitivity of 75%, specificity of 71% and AUC of 79% [59–99%] (CI: 0.95, p = 0.035). The best AUC was achieved by combining FDR%pred. with blood eosinophils count. At cut off values for FDR of 216%pred, and blood eosinophils of 365/μL, the ROC curve showed a sensitivity of 75%, specificity of 87%, and AUC of 85% [67–100%], (CI = 0.95, p = 0.01) (Fig. 1).

**Table 1:** Clinical characteristics of Responders and Partial-/Non-Responders at baseline and under treatment with Anti-T2 biological therapy

Variable	Baseline	Under treatment	Mean of difference	Percentage change (%)	P- Value
**Responders (n = 13)**					
Age (years)	59.4 ± 9.8				
Sex (f/m)	(9/4)				
BMI (kg/m^2^)	26.4 ± 6.1				
Blood eosinophil count (/μL)	620.8 ± 378.8	97.7 ± 160.7	− 523.1	− 84	< 0.01
FEV_1_(l)	2.1 ± 0.88	2.6 ± 1.1	+ 0.520	+ 24	< 0.01
FEV%	75.8 ± 21.7%	96.2 ± 18.1%	+ 20.4%	+ 26	< 0.01
FEF_25–75_ (l/s)	1.03 ± 0.77	1.64 ± 1.06	+ 0.61	+ 59	< 0.01
RV%	153 ± 21%	121 ± 20%	− 32%	− 21	0.02
RV/TLC	48 ± 9%	38 ± 9%	− 10%	− 21	0.02
FDR (KPa/l/s)	0.26 ± 0.20	0.12 ± 0.16	− 0.13	− 50	< 0.01
FDR%pred	270% ± 151	168% ± 85	− 103%	− 37	0.02
FeNo(ppb)	56.1 ± 26.4	40.8 ± 27.5	− 15.31	− 27	< 0.01
ACT score	14.2 ± 5.3	19.8 ± 4.7	+ 5.5	+ 39	< 0.01
Number of exacerbations	5.5 ± 3.1	1.1 ± 1.2	− 4.4	− 80	< 0.01
OSC dose (mg)	5.9 ± 5	0.4 ± 0.9	− 5.5	− 93	< 0.01
**Partial-/non-responders (n = 7)**					
Age (years)	60.86 ± 8.30				
Sex (f/m)	(3/4)				
BMI (kg/m^2^)	29.86 ± 3.0				
Blood eosinophil count (/μL)	474.3 ± 420.1	200.0 ± 355.9	− 274.2	− 58	> 0.05
FEV_1_ (l)	2.0 ± 0.58	1.7 ± 0.41	− 0.343	− 17	> 0.05
FEV%	77.6 ± 22.7%	67.1 ± 20.5%	− 10.4%	− 13	> 0.05
FEF_25–75_ (l/s)	1.13 ± 0.72	0.81 ± 0.45	− 0.32	− 28	> 0.05
RV%	140 ± 29%	150 ± 29%	+ 10%	+ 7	> 0.05
RV/TLC	45 ± 8%	50 ± 3%	+ 5%	+ 11	> 0.05
FDR (KPa/l/s)	0.13 ± 0.11	0.23 ± 0.17	+ 0.10	+ 76	> 0.05
FDR%pred	146% ± 76%	209% ± 151%	+ 63%	+ 43	> 0.05
FeNo (ppb)	41.4 ± 43.7	57.9 ± 38.7	+ 16.4	+ 39	> 0.05
ACT score	17.9 ± 4.9	17.3 ± 3.6	− 0.571	− 3	> 0.05
Number of exacerbations	4.1 ± 2.9	3.0 ± 2.0	− 1.14	− 27	> 0.05
OSC dose (mg)	7.2 ± 5.1	4.7 ± 3.7	− 2.6	36	> 0.05

Values at baseline and under treatment, as well as means of differences (+ or -) are presented as means ± standard deviations

Absolute blood eosinophils (cells/µl), FEV1: forced expiratory volume in the 1 s (l), FEF_25-75_: forced expiratory flow between 25 and 75% of the forced vital capacity, RV: residual volume, TLC: total lung capacity, FDR (%): frequency dependence resistance (R5-20 kPa/l/s) and predicted values, FeNO: fractional exhaled nitric oxide (ppb), ACT: asthma control test score, number of exacerbations in the last 12 months, OCS: oral corticosteroid (mg)

P values according to an appropriate test (t. test or Wilcoxon test). P-values in the last column represent test of baseline data vs. data under anti-T2 biological treatment within each of the two groups (responders vs. partial/non-responders). At baseline, clinical variables were also tested between groups and did not show any significant differences, except for FDR%pred (p = 0.038)

**Table 2:** Area under the curve (AUC) of clinical predictors

Clinical marker	AUC	P-value	95% Confidence Interval	
			Lower limit	Upper limit
FDR%	0.79	0.03	0.59	0.99
FeNO	0.76	0.06	0.49	1.00
Blood eosinophils	0.64	0.31	0.36	0.93
FEV1%	0.44	0.67	0.14	0.74
FeNO and blood eosinophils	0.72	0.10	0.46	0.99
FEV1 and blood eosinophils	0.66	0.23	0.39	0.94
FDR and blood eosinophils	0.85	0.01	0.67	1.00

Areas under the curves from (Fig. 1) used to compare the accuracy of each tested marker. P-Values and confidence intervals to define significant test (p > 0.05)

**Fig. 1:**
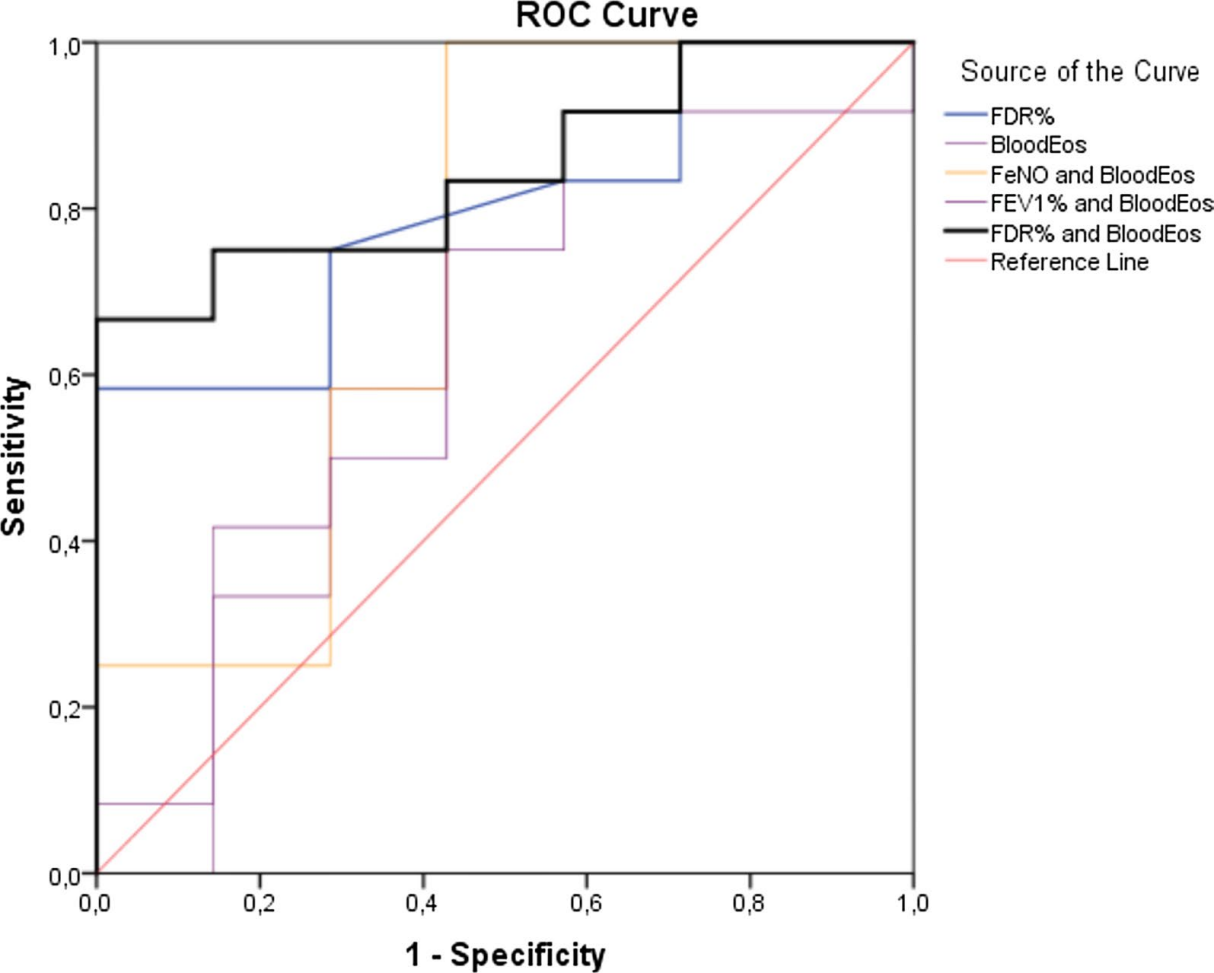
Receiver Operator Characteristic (ROC) curve: x axis (1-specifecity), y-axis sensitivity, each curve represents the predictability of each clinical marker (in different colors). AUC values and p-values for the separate variables are detailed in Table 2. A combination of FDR%pred. with absolute blood eosinophil counts demonstrated the best predictive capacity (AUC = 0.85, p-value = 0.01, CI95 = 0.67–1.00)

Within response groups, responders to anti-T2 biological therapies experienced significant improvements in all clinical markers (i.e. lung function, inflammatory biomarkers) in contrast to partial/non-responders that did not demonstrate any statistically significant changes; details are given in (Table 1).

Linear regressions indicated better associations between improvements in FDR and ACT score than with FEV1 and ACT score as well as better associations between improvements in FDR and reduction of exacerbations than with FEV1 (Fig. 2). The other included lung function measures (FEF_25–75_, RV and RV/TLC) were not superior to FEV1 when correlated to improvements in exacerbations or ACT with R^2^ values around 0.20.

**Fig. 2:**
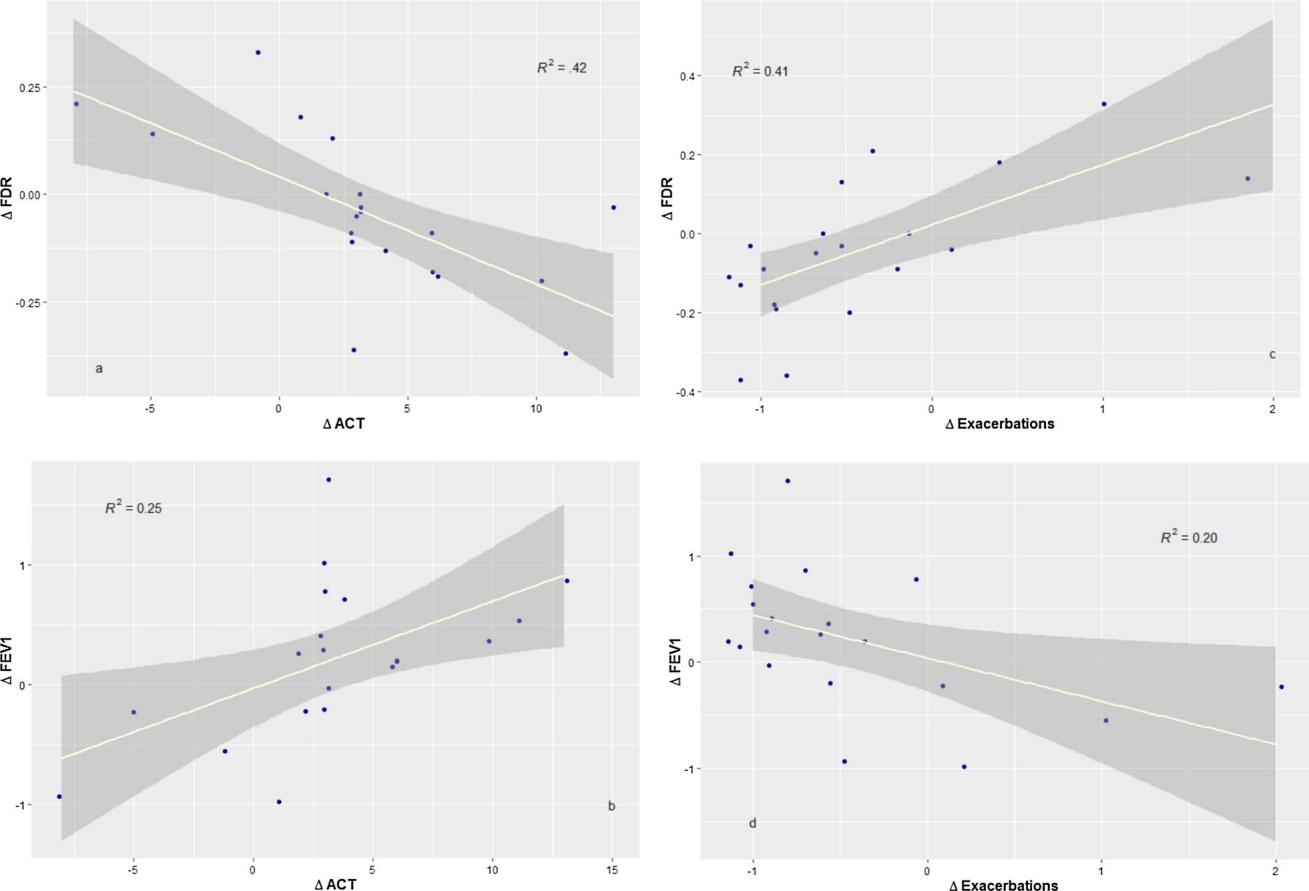
comparative linear regressions: between small airway dysfunction (FDR, KPa/L/s) and airflow limitation (FEV1, ml) in correlation to ACT score (points) and reduction in exacerbations (%). All values represent the difference (delta) between baseline and under therapy. A: linear regression between (delta ACT) scores on the x-axis, and the change in FDR (delta R5-20), y-axis: R^2^ = 0.42, DF = 18, p = 0.001. B: linear regression between (delta ACT) scores on the x-axis and the change in FEV1 (delta FEV1), y-axis: R^2^ = 0.25, DF = 18, p = 0.013. C: linear regression between (delta exacerbations) on the x-axis and the change in FDR (delta FEV1), y-axis: R^2^ = 0.41, DF = 18, p = 0.001. D: linear regression between (delta exacerbations) on the x-axis and the change in FEV1 (delta R5-20), y-axis: R^2^ = 0.20, DF = 18, p = 0.025

The main finding of our study is that SAD improves substantially under anti-T2 biological therapy in patients with severe eosinophilic asthma. Furthermore, pre-treatment IOS measures of SAD demonstrated to be meaningful predictors of clinical response, thereby indicating that severe SAD might describe a distinct phenotype with therapeutic implications among patients with severe eosinophilic asthma. Our results are consistent with the findings of previous studies which indicated that measures of SAD like FEF_25-75_, lung clearance index, regional ventilation inhomogeneity in acinar and conducting airways improve significantly under biological therapy [20, 21]. Oscillometric measures of SAD seem to be feasible tools in selecting appropriate patients qualifying for anti-T2 biological therapy beyond the rather crude measurement of baseline blood eosinophils count that is frequently influenced by a multitude of factors, e.g. dose of inhaled or oral steroids [22], diurnal variations [23] and atopic comorbidities [24]. Our observations on the improvements in SAD might be explained by the findings that in severe asthmatics, small airway function is significantly altered with type-2 inflammation (T cells and activated eosinophils infiltration) compared to large airways [25, 26]. In our study, partial or non-responders experienced a 58% mean reduction of circulating blood eosinophils (p > 0.05), indicating the pharmacological effect of the biological therapy without marked improvements in clinical symptoms. These results are consistent with the finding of Kelly et al., which showed that despite the clear reduction in circulating and airway eosinophilia under mepolizumab, it does not suppress other eosinophil activation markers which might explain the clinical deterioration in some subjects [27].

The main limitations of this study are its observational nature and the small number of the included subjects. Further, additional measures of small airway function like inert gas distribution could have supported our findings. Therefore, clinical trials involving larger cohorts and multimodular assessment of small airway function are needed to confirm our observation. Finally, two patients had a treatment with either dupilumab or benralizumab which do not share the exact same pharmacological mechanism compared with mepolizumab.

Our data support that SAD is potentially linked to asthma control as SAD improves substantially under anti-T2 biological therapy in therapy responders. Measures of small airway function like the FDR might be useful in selecting appropriate patients qualifying for anti-T2 biological therapy in addition to blood eosinophil count and might also serve as markers to assess clinical treatment response in patients with newly initiated biological therapy.

## Data Availability

The datasets used during the current study are available from the corresponding author on reasonable request.

## Abbreviations

ACT: Asthma control test
AUC: Area under the curve
BMI: Body mass index
DF: Degrees of freedom
FDR: Frequency dependence of resistance
FeNO: Fractional exhaled nitric oxide
FEV1: Forced expiratory volume in 1 s
IOS: Impulse oscillometry
LABA: Long-acting β_2_ adrenoceptor agonists
LAMA: Long-acting muscarinic receptor antagonists
MCID: Minimal clinically important difference
OCS: Oral corticosteroids
ROC: Receiver operator characteristic
RV: Residual volume
SAD: Small airways dysfunction
Th2: T helper 2 cells
TLC: Total lung capacity
